# Growth, Structure and Spectroscopic Characterization of Nd^3+^-Doped KBaGd(WO_4_)_3_ Crystal with a Disordered Structure

**DOI:** 10.1371/journal.pone.0040229

**Published:** 2012-07-06

**Authors:** Bin Xiao, Yisheng Huang, Lizhen Zhang, Zhoubin Lin, Guofu Wang

**Affiliations:** 1 Key Laboratory of Optoelectronics Material Chemistry and Physics, Fujian Institute of Research on the Structure of Matter, Chinese Academy of Sciences, Fuzhou, Fujian, China; 2 Graduate School of Chinese Academy of Sciences, Beijing, China; University of Akron, United States of America

## Abstract

The undoped and the Nd^3+^:KBaGd(WO_4_)_3_ crystals were grown by the top seeded solution growth (TSSG) method from a flux of K_2_W_2_O_7_. The structure of the pure crystal was determined by the single-crystal X-ray diffraction method. It crystallizes in the monoclinic symmetry with space group *C*2/*c*. In the structure, K^+^ and Ba^2+^ ions share the same 8*f* site with occupancy of 0.464 and 0.536, respectively. The investigation of spectral properties of Nd^3+^:KBaGd(WO_4_)_3_ crystal indicates that it exhibits broad absorption and emission bands, which are attributed to locally disordered environments around the Nd^3+^ centers. The broad absorption band is suitable for diode laser pumping.

## Introduction

The diode-laser pumped solid-state lasers are useful in a wide variety of applications in the fields of military [1], [2], industry [3] and medical treatments [4] due to their advantages, such as outstanding stability, high efficiency, compact size and long lifetime. A lot of well-known Nd^3+^-doped laser crystals (Nd:YAG, for example [5]) are commercially available, however, they can be hardly pumped with diode-lasers. Because they have very narrow absorption bands near their pumped wavelength compared to the emission bandwidths of common diode-lasers (several nanometers), they cannot absorb the pump energy of diode-lasers efficiently. Besides, the temperature stability of the emission wavelength of diode-lasers needs to be crucially controlled for laser crystals with narrow absorption bands, since the emission wavelength of the diode-lasers changes at 0.2–0.3 nm/K with the operating temperature of the laser device. [6] As a consequence, it is necessary to explore novel laser crystals with large absorption bandwidths for diode pumping.

The broadening of the spectral features can be expected for solid-state materials with multisites, defects, or local disorder [7]. In the past few years, there have been a lot of studies on the family of scheelite-related disordered molybdate and tungstate crystals AT(XO_4_)_2_ (A = monovalent Li, Na; T = trivalent Bi, Y, La-Lu; X = Mo, W) [8]–[10]. These disordered crystals have been demonstrated as promising materials in the domains of tunable and ultrashort femto-second laser pulse due to the obvious advantage of large spectral broadening. Recently, we synthesized a new triple tungstate KBaGd(WO_4_)_3_ compound also with disordered structure. Because of the mixed K(Ba) occupancy of the same crystallographic site, 8*f*, the structure of KBaGd(WO_4_)_3_ presents some local disorder around Gd^3+^ sites, and thus can lead to the spectral broadening when rare-earth luminescent ions (such as Nd^3+^) replace Gd^3+^. Therefore, this paper reports on the growth, structure and spectral properties of Nd^3+^-doped KBaGd(WO_4_)_3_ crystal.

## Materials and Methods

### 1.Synthesis and Crystal Growth

Polycrystalline samples of undoped KBaGd(WO_4_)_3_ were synthesized by means of the solid-state reaction method. The synthesis process followed that of ref. [11]. To evaluate melting point of the synthesized KBaGd(WO_4_)_3_ compound, the thermal analysis was carried out by differential scanning calorimetry (DSC) using a NETZCH STA 499C Simultaneous Thermal Analyzer, which was performed up to 1563 K at a heating and cooling rate of 15 K min^−1^.

**Figure 1:**
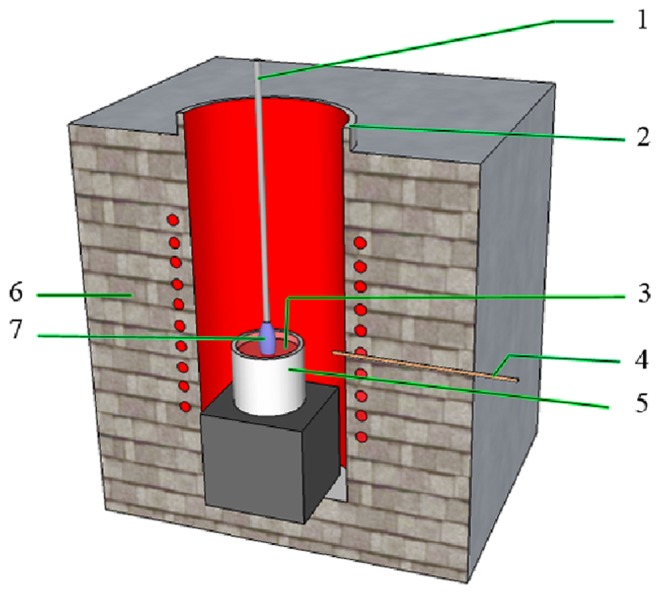
Schematic diagram of crystal growth apparatuses: (1) seed holder; (2) Al_2_O_3_ tube; (3) melt; (4) thermocouple; (5) crucible; (6) thermal insulation material; (7) seed.

The undoped and Nd^3+^:KBaGd(WO_4_)_3_ crystals were grown by the top seeded solution growth method. The crystal growth was carried out in a vertical tubular furnace, as shown in Fig. 1. An AL-708 controller with controlling accuracy of ±0.1 K was used to control the furnace temperature and the cooling rate.

### 2.Crystal Structure Analysis

A single crystal of undoped KBaGd(WO_4_)_3_ with dimensions of 0.10×0.10×0.10 mm^3^ was selected for X-ray diffraction determinations. The diffraction data were collected on a Mercury70 CCD diffractometer equipped with graphite-monochromated Mo Kα (λ = 0.71073 Å) radiation at 293 K. A total of 1214 independent reflections were collected in the range of 3.35° < θ <27.45°, of which 1102 with *I* ≥*2σ(I)* were independent. The absorption correction based on the empirical PSI-scan technique was applied. The structure was solved by direct methods and refined by the full-matrix least squares technique with the SHELXL97 program package [12]. The final unweighted residual factor *R* which is used to judge the structure model is 0.0229. The weighted residual factor *wR* is 0.0514 with the weighting factor *w*:
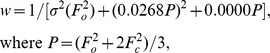
in the above equations, *σ*(*F_o_* ^2^) is the estimated standard uncertainty of the observed reflections, *F_o_* ^2^ and *F_c_* ^2^ means the observed and calculated squared structure factors, respectively. After the refinement, the residual electron density (Δρ)_max = _1.542, and (Δρ)_min_^ = ^−2.045 e/Å^3^, (Δ/σ)_max_ = 0.000. The value of goodness of fit *S* which is used to adjust the weighting scheme is 1.050. The details of the X-ray structure analysis are listed in (Table S1). The X-ray power diffraction (XRD) pattern of undoped KBaGd(WO_4_)_3_ was determined using a D-max-rA type diffractometer with Cu Kα radiation (λ = 1.54056 Å) at room temperature. The power electron diffraction pattern of undoped KBaGd(WO_4_)_3_ crystal which was carried out by a JEM-2010 transmission electron microscope.

### 3.Spectral Characterization

**Figure 2:**
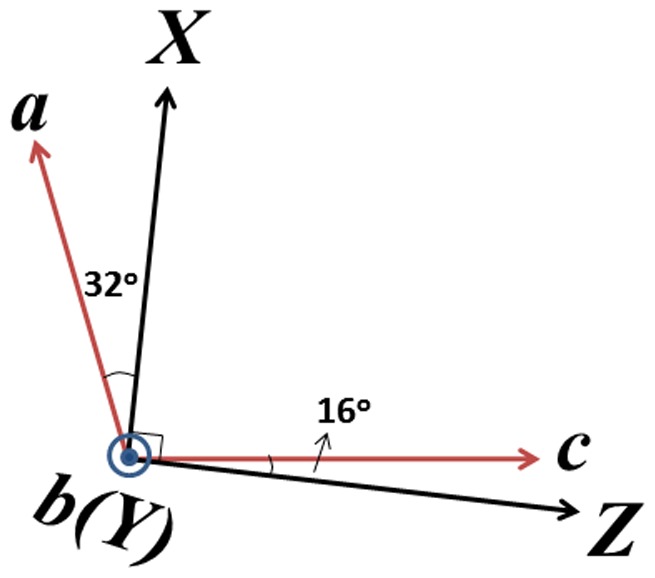
Relative orientation between the optical indicatrix axes (*X*, *Y*, *Z*) and the crystallographic axes (*a*, *b*, *c*) of the Nd^3+^:KBaGd(WO_4_)_3_ crystal. The plot means view from the positive *b*-axis direction.

**Figure 3:**
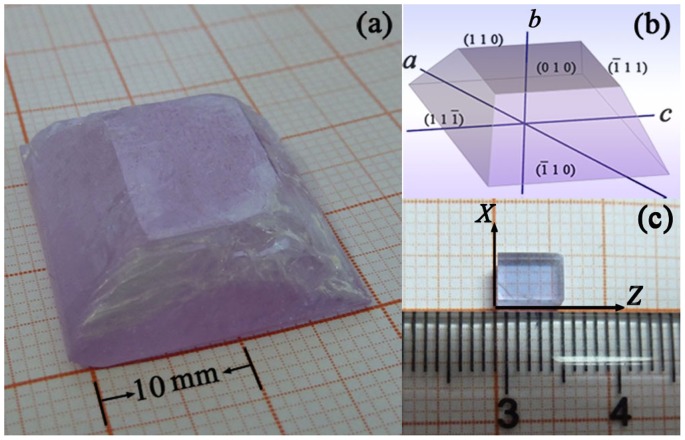
(a) Nd^3+^:KBaGd(WO_4_)_3_ crystal grown by TSSG method. (b) Stimulated facets marked by Miller indices (hkl). (c) An orientated rectangle sample cut from the as-grown crystal. Each face is perpendicular to one of the optical indicatrix axes.

Since Nd^3+^:KBaGd(WO_4_)_3_ crystal with the monoclinic system is optically biaxial, three optical indicatrix axes (*X*, *Y*, *Z*) should be determined before the measurement of polarized spectra. One of the optical indicatrix axes (*Y*) is parallel to the unique two-fold crystallographic axis (*b*-axis, which is also the *C*_2_ symmetry axis), and the other two lie in a perpendicular plane to this axis. The orientation of the crystal was performed by means of crossed polarized microscope, and the orientating procedure follows that of ref. [13]. The *c*-axis is at 16° anticlockwise with respect to the indicatrix *Z*-axis, and the angular relationship between the optical indicatrix axes and the crystallographic axes (*a*, *b*, *c*) for Nd^3+^:KBaGd(WO_4_)_3_ crystal is shown in Fig. 2. After orientation, a sample with dimensions of 5.5×3.8×2.6 mm^3^ and high optical quality was cut from the as-grown crystal, as shown in Fig.3 (c).

The polarized absorption spectra were measured with a Perkin-Elmer UV-VIS-NIR spectrophotometer (Lambda 900). Excited by a continuous Xe-lamp at 805 nm, the polarized emission spectra were recorded using an Edinburgh Instruments FLS920 spectrophotometer. All the spectral experiments were carried out at room temperature. In spectral experiment, the electric field of the light is parallel to each one of the three optical indicatrix axes, *E*||*X*, *E*||*Y*, and *E*||*Z.*

## Results and Discussion

### 1.DSC Analysis and Crystal Growth

**Figure 4:**
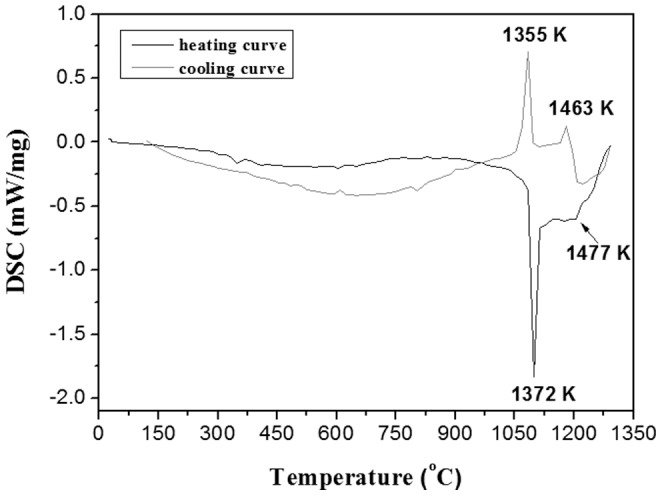
DSC curves of undoped KBaGd(WO_4_)_3_ compound.

The DSC result reveals that the undoped KBaGd(WO_4_)_3_ crystal melts incongruently (Fig. 4). In the heating cycle, an endothermic peak corresponding to a solid-liquid transformation is observed, which means the KBaGd(WO_4_)_3_ is decomposed at 1372 K, and then melts completely to form the liquid phase at 1477 K. The cooling curve shows two exothermic peaks with maxima at 1463 K and 1355 K, respectively, with the second one corresponding to resolidification of KBaGd(WO_4_)_3_.

Since the KBaGd(WO_4_)_3_ crystal exists a phase transition at temperatures lower than the melting point, hence the best technique to grow it is the top seeded solution growth method (TSSG) [14]. The Nd^3+^:KBaGd(WO_4_)_3_ crystal was grown in a flux of K_2_W_2_O_7_ by the this method, where the molar ratio of KBaGd(WO_4_)_3_ to K_2_W_2_O_7_ is 1∶1. The chemical used were K_2_CO_3_, BaCO_3_, WO_3_ with analytical-grade as well as Gd_2_O_3_ and Nd_2_O_3_ with 99.99% purity. According to their stoichiometric composition, 3.0 at % Nd^3+^-doped KBaGd(WO_4_)_3_ and K_2_W_2_O_7_ were weighed. The weighed materials were mixed and put in a platinum crucible with a volume of 100 mm^3^. The mixture was slowly heated up to 1273 K in air atmosphere, and then maintained at that temperature for 48 hours to achieve a homogeneous melt. The saturation temperature of the solution was determined by repeated seeding. The starting growth temperature was about 1203 K. The crystals were grown at a cooling rate of 1K/day and a rotating rate of 45 rpm. When the growth ended, the grown crystals were carefully withdrawn from the solution and cooled to room temperature at 15 K h^−1^. The Nd^3+^:KBaGd(WO_4_)_3_ crystal with few inclusions and dimensions of 21×24×12 mm^3^ was obtained, as shown in Fig. 3(a). The morphology of the crystal is shown in Fig. 3(b), which was simulated with Bravais-Friedel and Donnay-Harker based *WinXMorph* software. [15].

The composition of the grown crystal was measured in an inductively coupled plasma by atomic emission spectrometry (ICP-AES) technique. For this purpose, the Nd^3+^:KBaGd(WO_4_)_3_ sample was heated at 363 K and dissolved in 37% HCl, then the signal of ICP intensity was quantified from the comparison of the standard elements and corrected by the calibration curve. The result of ICP is listed in Table 1. The measured molar ratio K: Ba: (Gd+Nd): W is close to 1∶1∶1:3, which agrees with the composition of Nd^3+^:KBaGd(WO_4_)_3_ compounds.

**Table 1:** Summary of the elemental analysis results of 3.08 at% Nd^3+^-doped KBaGd(WO_4_)_3_ crystal by the ICP-AES technique.

				(Gd+Nd)^a^	
Elements	W	K	Ba	Gd	Nd
Required (Wt%)	51.22	3.63	12.75	14.02	0.54
Measured (wt%)	50.96±1.02	3.27±0.07	12.43±0.25	13.84±0.28	0.43±0.009
Calculated molar number (10^−2^)	27.72±0.55	8.36±0.18	9.05±0.18	8.80±0.18	0.30±0.006
Molar ratio	3.06±0.12	0.92±0.038	1±0.04	0.97±0.039	0.0309±0.0013
				(Gd+Nd) ≈ 1	

a the Gd^3+^ ions were generally substituted by Nd^3+^ ions in the Nd^3+^-doped KBaGd(WO_4_)_3_ crystal because the Gd^3+^ and Nd^3+^ ions have the same valence.

### 2.Crystal Structure

**Figure 5:**
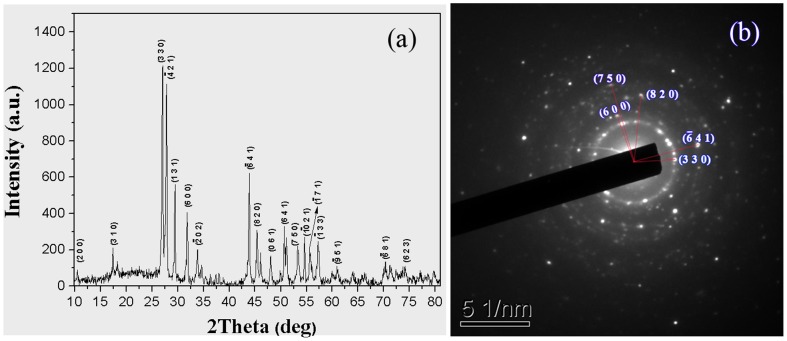
(a) Powder XRD patterns of undoped KBaGd(WO_4_)_3_ crystal. (b) Powder electron diffraction pattern of the undoped KBaGd(WO_4_)_3_ crystal.

Fig. 5(a) shows the XRD pattern of undoped KBaGd(WO_4_)_3_ crystal that can be indexed on the basis of lattice parameters. The indexed result of power electron diffraction pattern of undoped KBaGd(WO_4_)_3_ crystal is in agreement with that of the powder X-ray diffraction pattern, as shown in Fig. 5(b), which confirmed the XRD result. The powder XRD data of undoped KBaGd(WO_4_)_3_ crystal are listed in Table S2.

The crystallographic analysis of the undoped KBaGd(WO_4_)_3_ crystal reveals that it crystallizes in the monoclinic system with space group *C*2/*c*. The lattice parameters of KBaGd(WO_4_)_3_ crystal are: a* = *17.544(4) Å, *b = *12.1742(16) Å, *c = *5.3202(9) Å, *β = *105.498(11)°, and *V = *1095.0(3) Å^3^ (also see CIF file: CIF S1). The atomic coordinates and thermal parameters are given in Table S3. In the refined structure, the Gd and W(2) fully occupy 4*e* sites, and W(1) as well as the eight types of O are found in different 8*f* sites. After least-squares refinement, the valence charge equilibrium and the magnitude of temperature factors demonstrate the existence of a statistic distribution of K and Ba, in single 8*f* site with occupancy factors fixed to 0.464∶0.536, which shows that KBaGd(WO_4_)_3_ crystal has a high disordered structure.

**Figure 6:**
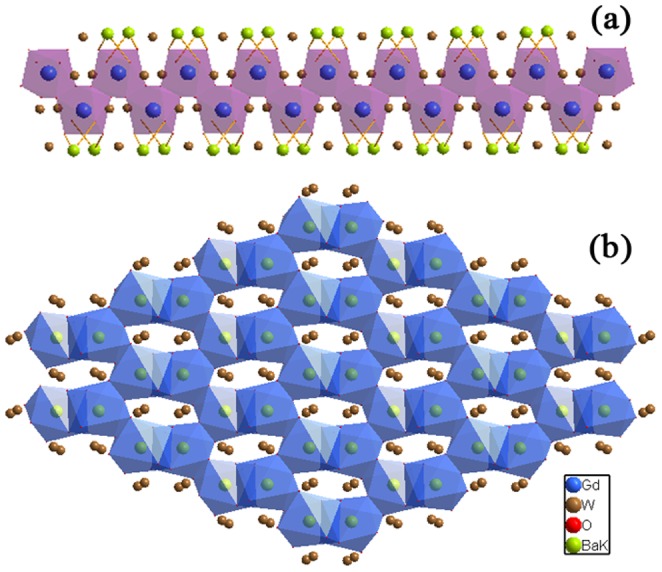
View of the *ab* plane of the KBaGd(WO_4_)_3_ crystal. (a) Chain formed by GdO_8_ disordered polyhedra. (b) Six-membered rings of square antiprisms K/BaO_8_.

**Figure 7:**
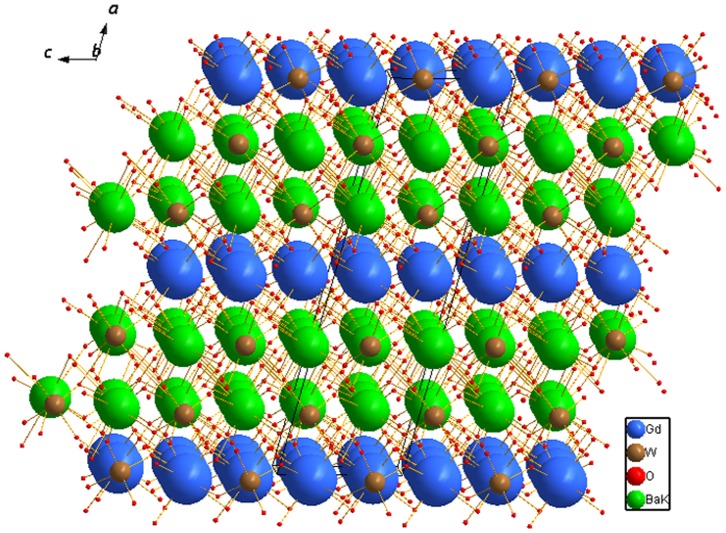
View along the *ac* plane shows the layers stacked along the *a*-axis.

The crystalline structure is constituted by K/BaO_8_ distorted square antiprisms with *C*_1_ symmetry, distorted GdO_8_ polyhedra which form chains lying along the *c*-axis (see Fig. 6 (a)) and two kinds of distorted WO_4_ tetrahedra. It can be described as layers stacked along the *a*-axis formed by corrugated six-membered rings of disordered K/BaO_8_ polyhedra, which share edges (Fig. 6 (b)). Adjacent layers are connected through distorted WO_4_ polyhedra and GdO_8_ chains (Fig. 7). The distinctive layered arrangement further increases the locally variable crystal field around Gd sites. The Gd^3+^ ions could provide appropriate sites for Nd^3+^ replacements because the Gd^3+^ and Nd^3+^ ions have the same valence, and additionally offer multiple distributions of cationic environments around these sites due to the coexistence of K^+^, Ba^2+^ and Gd^3+^ ions. Therefore, when Nd^3+^ replaces Gd^3+^ ions, the high disordered environment around the Nd^3+^ dopant ions leads to inhomogeneous broadening of the absorption and emission bands [16], [17].

### 3.Spectral Properties

**Figure 8:**
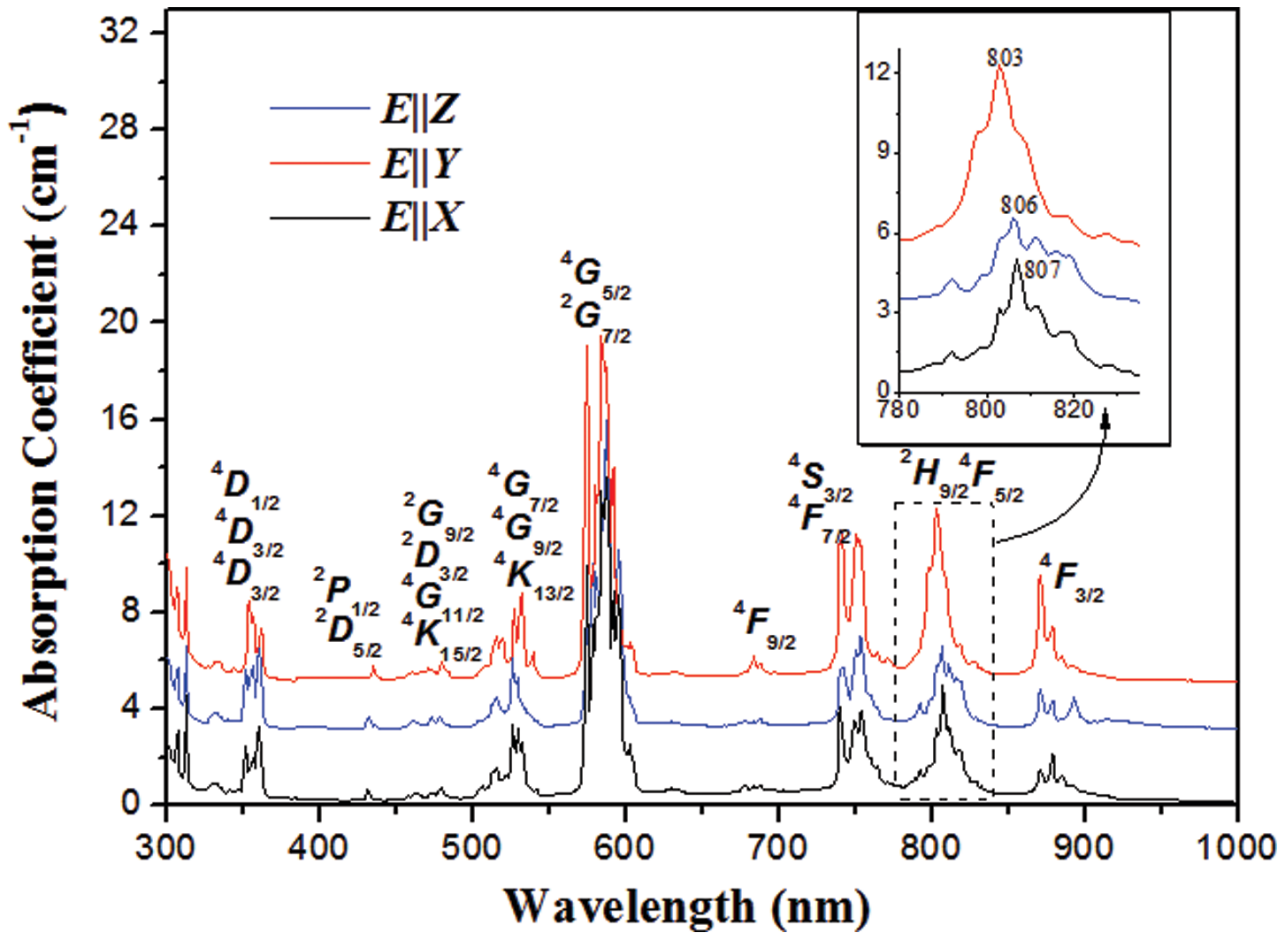
Polarized absorption spectra of Nd^3+^-doped KBaGd(WO_4_)_3_ crystal at 300 K. The inset shows the spectra in the range of 780 to 840 nm.

Fig. 8 shows the polarized absorption spectra of the Nd^3+^-doped KBaGd(WO_4_)_3_ at room temperature. These absorption lines are due to transition from the ground state ^4^ *I*_9/2_ to the various excited states of Nd^3+^ ions. In the absorption spectra, the most interesting is the broad absorption bands in the range of 780–840 nm, which is close to the output wavelength of commercially diode laser devices. The absorption spectra are strong polarization dependent because of the anisotropy of the monoclinic crystal. The absorption spectrum for *E*||*Y* polarization is strongest among the three absorption spectra and it has a full-width at half-maximum (FWHM) of 14 nm at 803 nm, which is larger than that of the ordered crystal, like Nd^3+^:BaGd_2_(MoO_4_)_4_ crystal (4 nm, *E*||*Z*) or LaB_3_O_6_ (5 nm, *E*||*X*) [18], [19]. Such large FWHM caused by the highly disordered structure around Nd^3+^ centers is suitable for diode-laser pumping. This demonstrates the Nd^3+^-doped KBaGd(WO_4_)_3_ crystal can be pumped effectively and not restricted to the temperature stability of the output wavelength of diode-laser.

The absorption cross-section σ*_a_* was calculated using σ*_a_ = *α/*N*_c_ formula, where α is the absorption coefficient and *N*_c_ is the concentration of Nd^3+^. The concentration of Nd^3+^ ions in Nd^3+^:KBaGd(WO_4_)_3_ crystal was measured to be 3.08 at% (i.e. 1.12 ×10^−20^ cm^−3^), and the results of the σ*_a_* are listed in Table 2.

**Table 2:** Comparison of spectral parameters of Nd^3+^:KBaGd(WO_4_)_3_ with other Nd^3+^-doped laser crystals.

Crystal	Polarization	Absorption(∼805nm)		Emission (∼1060 nm)		Disordered	Ref.
		FWHM	σ*_a_*	FWHM	σ*_e_*		
		(nm)	(10^−20^cm^2^)	(nm)	(10^−20^ cm^2^)		
KBaGd(WO_4_)_3_	*E*||*X*	11	4.5	19	4.5		
	*E*||*Y*	14	6.9	24	6.5	Yes	
	*E*||*Z*	19	4.1	19	4.1		
							
LiLa(WO_4_)_2_	*π*-polarization	20	2.2	–	8.9	Yes	[24]
	*σ*-polarization	20	2.8	–	7.8		
							
NaY(WO_4_)_2_	*π*-polarization	17	–	≈ 13	–	Yes	[25]
	*σ*-polarization	17	–	≈ 13	–		
KBaGd(MoO_4_)_3_	unpolarized	9	11.1	24	5.17	Yes	[16]
							
BaGd_2_(MoO_4_)_4_	*E*||*X*	–	4.3	4	8.6		
	*E*||*Y*	–	3.3	4	7.2	No	[18]
	*E*||*Z*	4	17.5	3	19.2		
							
LaB_3_O_6_	*E*||*X*	3	5.0	4	10.2		
	*E*||*Y*	3	4.5	3	8.3	No	[19]
	*E*||*Z*	4	5.5	4	7.2		

The emission cross-sections were calculated using the Füchtbauer-Ladenbrug (F-L) equation, [20].
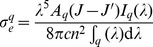
(1)


where *I_q_*(*λ*) is the emission intensity at wavelength *λ* with *q* polarization, *A_q_* is the emission probability which can be calculated from the Judd-Ofelt theory [21], [22], the refractive index *n* is calculated to be 1.83 using the method mentioned in Ref. [23].

**Figure 9:**
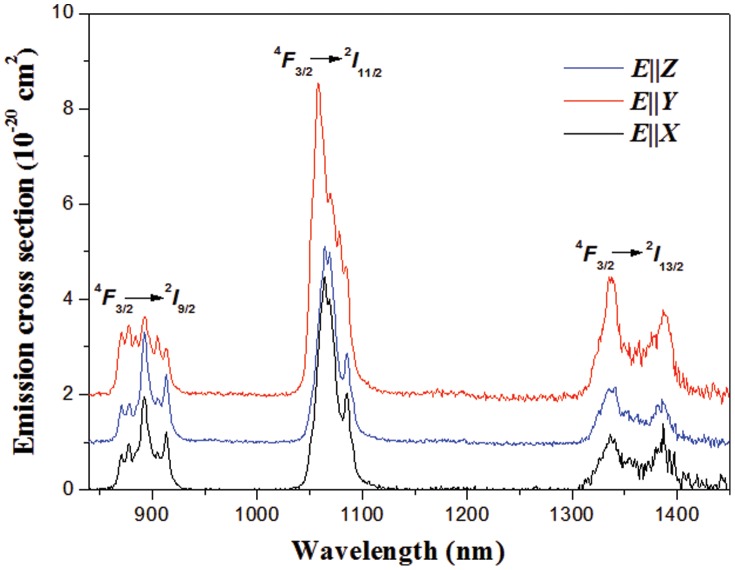
Polarized emission spectra of Nd^3+^-doped KBaGd(WO_4_)_3_ crystal excited with 805 nm radiation at 300 K.

Fig. 9 shows the wavelength dependences of the stimulated emission cross-sections. Three emission bands observed at 850–950, 1020–1150 and 1300–1450 nm are due to the transitions of ^4^ *F*_3/2_→^4^ *I*_9/2_, ^4^ *F*_3/2_→^4^ *I*_11/2_ and ^4^ *F*_3/2_→^4^ *I*_13/2_, respectively. The most important transition of ^4^ *F*_3/2_→^4^ *I*_11/2_ is centered at about 1060 nm. The FWHM for *E*||*Y* polarization is 24 nm, which is much larger than that of ordered structure crystals (see Table 2). The peak stimulated emission cross-sections for the transition of ^4^ *F*_3/2_→^4^ *I*_11/2_ are 4.5, 6.5 and 4.1×10^−20^ cm^2^ for *E*||*X*, *E*||*Y* and *E*||*Z*, respectively.

### 4.Conclusion

The 3.08 at% Nd^3+^-doped KBaGd(WO_4_)_3_ crystal was successfully grown by the TSSG method. The thermal analysis shows it melts incongruently at 1372 K. The structure analysis indicates that the undoped KBaGd(WO_4_)_3_ crystal has a statistic distribution of K(0.464) and Ba(0.536) atoms. Each Ba(K) is coordinated by eight oxygen atoms to form a distorted polyhedron. This fact and its layered arrangement structure lead to locally disordered environments around the Gd^3+^ ions. When Gd^3+^ ions are substituted by Nd^3+^ dopant ions, the disordered structure results the broad FWHM of absorption and emission bands. Such broad FWHM of absorption band is suitable for diode-laser pumping. The Nd^3+^:KBaGd(WO_4_)_3_ crystal with broad FWHM of emission band is promising as a candidate for tunable and short pulse solid-state laser operation. The spectroscopic properties of the Nd^3+^:KBaGd(WO_4_)_3_ crystal are anisotropic with the largest absorption and emission cross sections for *E*||*Y* polarization. The Nd^3+^:KBaGd(WO_4_)_3_ crystal has large absorption and emission cross-sections, which are 6.9×10^−20^ cm^2^ at 805 nm and 6.5×10^−20^ cm^2^ at 1060 nm respectively.

## Supporting Information

Table S1 **Crystal data and structure refinement details for undoped KBaGd(WO_4_)_3._**
(DOC)

Table S2 **Powder XRD data of undoped KBaGd(WO_4_)_3_ crystal.**
(DOC)

Table S3 **Atomic coordinates and thermal parameters for undoped KBaGd(WO_4_)_3_ crystal.**
(DOC)

CIF S1 **The CIF file of the undoped KBaGd(WO_4_)_3_ crystal.**
(CIF)
