# Supplementary material for: Growth, Structure and Spectroscopic Characterization of Nd3+-Doped KBaGd(WO4)3 Crystal with a Disordered Structure
Source: PLoS One. 2012 Jul 6;7(7):e40229. doi: 10.1371/journal.pone.0040229 (PMC3391210; doi:10.1371/journal.pone.0040229)
Supplement: Table S1 — Crystal data and structure refinement details for undoped KBaGd(WO4)3. (DOC) [file pone.0040229.s001.doc]

| empirical formula | KBaGd(WO_4_)_3_ |
| --- | --- |
| formula weight | 1077.24 |
| temp. (K), wavelength (Å) | 293(2), 0.71073 |
| crystal system, space group | monoclinic, *C*2/*c* |
| unit cell dimensions |  |
| *a* (Å) | 17.544(4) |
| *b* (Å) | 12.1742(16) |
| *c* (Å) | 5.3202(9) |
| *β* (deg) | 105.498(11) |
| *V*(Å^3^), *Z* | 1095.0(3), 4 |
| *D*_cal_ (g cm^-3^) | 6.535 |
| *μ* (mm^-1^) | 41.348 |
| *F*(000) | 1828 |
| crystal size (mm) | 0.10 × 0.10 × 0.10 |
| theta range (deg) | 3.35 − 27.45 |
| index ranges | *-*22 *≤ h ≤* 22 |
|  | -15 *≤ k ≤* 15 |
|  | -6 *≤ l ≤* 6 |
| reflections collected | 3938 |
| independent reflections | 1214 [*R*_int_ = 0.0319] |
| refinement method | Full-matrix least-squares on *F*^2^ |
| goodness-of-fit on *F*^2^ | 1.050 |
| final *R* indices [*I* > 2*σ*(*I*)] | *R*_1_ = 0.0229, *wR*_2_ = 0.0514 |
| *R* indices (all data) | *R*_1_ = 0.0266, *wR*_2_ = 0.0534 |
| largest diff. peak and hole (e Å^‑3^) | 1.542 and -2.045 |
