# Supplementary material for: Growth, Structure and Spectroscopic Characterization of Nd3+-Doped KBaGd(WO4)3 Crystal with a Disordered Structure
Source: PLoS One. 2012 Jul 6;7(7):e40229. doi: 10.1371/journal.pone.0040229 (PMC3391210; doi:10.1371/journal.pone.0040229)
Supplement: Table S2 — Powder XRD data of undoped KBaGd(WO4)3 crystal. (DOC) [file pone.0040229.s002.doc]

| h k l | 2θ | d_obs_(Å) | d_cal_ (Å) | I/I_0_ |  | h k l | 2θ | d_obs_ (Å) | d_cal_ (Å) | I/I_0_ |
| --- | --- | --- | --- | --- | --- | --- | --- | --- | --- | --- |
| 1 1 0 | 9.032 | 9.7833 | 9.8793 | 9.6 |  | 4 6 0 | 49.932 | 1.825 | 1.8292 | 4.9 |
| 2 0 0 | 10.502 | 8.4169 | 8.4531 | 5.7 |  | 6 4 1 | 50.676 | 1.7999 | 1.7991 | 26.9 |
| 3 1 0 | 17.419 | 5.0869 | 5.114 | 12.5 |  | 7 3 1 | 51.144 | 1.7845 | 1.7864 | 20.6 |
| 3 3 0 | 27.042 | 3.2946 | 3.2931 | 100 |  | 7 5 0 | 53.311 | 1.717 | 1.7147 | 17.2 |
| 2 1 | 27.797 | 3.2068 | 3.2216 | 95.1 |  | 2 1 | 54.607 | 1.6792 | 1.6825 | 19.1 |
| 1 3 1 | 29.444 | 3.031 | 3.0368 | 45.8 |  | 7 1 | 55.616 | 1.6512 | 1.653 | 14.2 |
| 6 0 0 | 31.818 | 2.8101 | 2.8177 | 33.9 |  | 3 3 | 57.254 | 1.6077 | 1.6051 | 19.3 |
| 0 2 | 33.838 | 2.6469 | 2.6582 | 14.9 |  | 2 2 3 | 59.905 | 1.5428 | 1.5423 | 4.2 |
| 3 3 1 | 34.635 | 2.5878 | 2.5928 | 5.5 |  | 5 1 | 61.094 | 1.5156 | 1.5168 | 5.1 |
| 0 2 2 | 38.007 | 2.3655 | 2.3624 | 4.8 |  | 8 1 | 63.898 | 1.4557 | 1.4563 | 5.2 |
| 4 1 | 43.861 | 2.0624 | 2.063 | 53.3 |  | 12 0 0 | 66.269 | 1.4092 | 1.4088 | 5.2 |
| 8 2 0 | 45.401 | 1.996 | 1.9964 | 24.9 |  | 1 1 | 70.063 | 1.3419 | 1.3412 | 6.9 |
| 4 0 2 | 46.102 | 1.9673 | 1.9707 | 12.8 |  | 8 1 | 70.348 | 1.3372 | 1.3377 | 8.5 |
| 0 6 1 | 48.046 | 1.8921 | 1.8866 | 13.4 |  | 1 4 | 71.204 | 1.3232 | 1.3211 | 6.4 |
