# Supplementary material for: Growth, Structure and Spectroscopic Characterization of Nd3+-Doped KBaGd(WO4)3 Crystal with a Disordered Structure
Source: PLoS One. 2012 Jul 6;7(7):e40229. doi: 10.1371/journal.pone.0040229 (PMC3391210; doi:10.1371/journal.pone.0040229)
Supplement: Table S3 — Atomic coordinates and thermal parameters for undoped KBaGd(WO4)3 crystal. (DOC) [file pone.0040229.s003.doc]

| Atom | Site | x | y | z | *U*eq(Å^2^) |
| --- | --- | --- | --- | --- | --- |
| Gd | 4e | 0.0000 | 0.62012(4) | 0.2500 | 0.00631(13) |
| W1 | 8f | 0.157124(17) | 0.60735(2) | -0.10030(5) | 0.00763(12) |
| W2 | 4e | 0.0000 | 0.85149(4) | -0.2500 | 0.00819(13) |
| O1 | 8f | -0.0402(2) | 0.7711(4) | -0.0320(9) | 0.0118(10) |
| O2 | 8f | 0.2275(3) | 0.5284(4) | 0.1301(9) | 0.0156(11) |
| O3 | 8f | 0.1103(3) | 0.6883(4) | 0.0954(9) | 0.0112(10) |
| O4 | 8f | 0.0776(3) | 0.9351(4) | -0.0707(9) | 0.0176(11) |
| O5 | 8f | 0.0769(2) | 0.5378(4) | 0.6534(9) | 0.0116(10) |
| O6 | 8f | 0.2929(3) | 0.8020(4) | 0.2553(9) | 0.0123(10) |
| Ba | 8f | 0.16553(3) | 0.87506(5) | 0.42237(12) | 0.0116(2) |
| K | 8f | 0.16553(3) | 0.87506(5) | 0.42237(12) | 0.0116(2) |
